# Investigation of the Affinity of Ceftobiprole for Selected Cyclodextrins Using Molecular Dynamics Simulations and HPLC

**DOI:** 10.3390/ijms242316644

**Published:** 2023-11-23

**Authors:** Dariusz Boczar, Katarzyna Michalska

**Affiliations:** Department of Synthetic Drugs, National Medicines Institute, Chełmska 30/34, 00-725 Warsaw, Poland; d.boczar@nil.gov.pl

**Keywords:** ceftobiprole, cyclodextrin, β-CD, HP-β-CD, SBE-β-CD, inclusion complex, molecular dynamics, GBSA

## Abstract

This paper presents the theoretical calculations of the inclusion complex formation between native ceftobiprole, a promising antibiotic from the cephalosporin group, and selected cyclodextrins (CDs) approved by the European Medicines Agency. Ceftobiprole was studied in three protonation states predicted from pK_a_ calculations, along with three selected CDs in a stoichiometric ratio of 1:1. It was introduced into the CD cavity in two opposite directions, resulting in 18 possible combinations. Docking studies determined the initial structures of the complexes, which then served as starting structures for molecular dynamics simulations. The analysis of the obtained trajectories included the spatial arrangement of ceftobiprole and CD, the hydrogen bonds forming between them, and the Gibbs free energy (ΔG) of the complex formation, which was calculated using the Generalised Born Surface Area (GBSA) equation. Among them, a complex of sulfobutyl ether- (SBE-) β-CD with protonated ceftobiprole turned out to be the most stable (ΔG = −12.62 kcal/mol = −52.80 kJ/mol). Then, experimental studies showed changes in the physiochemical properties of the ceftobiprole in the presence of the CDs, thus confirming the validity of the theoretical results. High-performance liquid chromatography analysis showed that the addition of 10 mM SBE-β-CD to a 1 mg/mL solution of ceftobiprole in 0.1 M of HCl increased the solubility 1.5-fold and decreased the degradation rate constant 2.5-fold.

## 1. Introduction

**Scheme 1:**
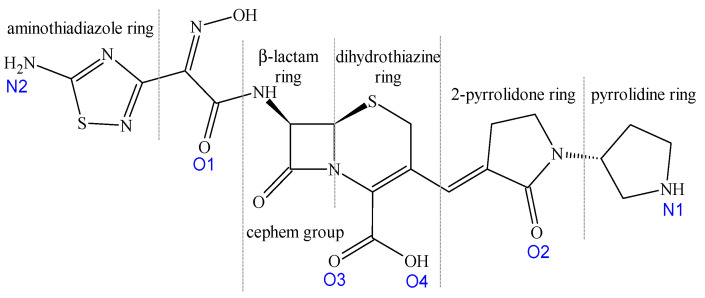
Molecular structure of ceftobiprole. The introduced atom labels are used in the analysis of hydrogen bonds.

Ceftobiprole (C_20_H_22_N_8_O_6_S_2_, Scheme 1) is an antibacterial agent belonging to the newest, fifth generation of cephalosporins, which is the major subgroup of semi-synthetic β-lactam antibiotics. It was approved and authorised in some countries in October 2018 as the prodrug ceftobiprole medocaril (C_26_H_26_N_8_O_11_S_2_) under the trade name of Zeftera© (previously Zevtera) or Mabelio©. Ceftobiprole was the first β-lactam to show in vitro activity against multidrug-resistant pathogens, such as methicillin-resistant *S. aureus* (MRSA), vancomycin-resistant *S. aureus* (VRSA), and penicillin-resistant *Streptococcus pneumoniae* (PRP) [1]. The activity against gram-negative bacteria corresponds to the known activity of other third-generation cephalosporins, such as ceftazidime. Considering the need to introduce new, effective antibacterial drugs into therapies that are active in the cases of pathogens with a severe clinical course in particular, ceftobiprole is an important alternative to empiric treatment in the cases of community-acquired pneumonia and non-ventilator-associated hospital-acquired pneumonia [2,3] when MRSA is proven or strongly suspected.

Native ceftobiprole is characterised by low solubility in water. In the registered medicinal product, this problem was solved by attaching a medocaril group, making the molecule more hydrophilic. The formation of a prodrug that is converted into an active drug in vivo is one of the methods for enhancing the water solubility and bioavailability of the drug; however, the efficiency of biotransformation may differ from person to person because it depends on individual metabolic characteristics. Moreover, a decline in biotransformation capacity is observed with age in humans [4]. Alternative methods of bioavailability enhancement include creating lipid-based drug delivery systems [5], obtaining a long-term stable amorphous solid dispersion of the drug with an excipient, prebiotic, or other drug [6], and forming a drug inclusion complex with cyclodextrin (CD) [7]. These methods do not involve the chemical derivatisation of an active substance to form a prodrug and do not rely on human biotransformation; therefore, in this approach, a novel delivery system of native ceftobiprole with selected CDs was proposed on the basis of theoretical methods.

**Scheme 2:**
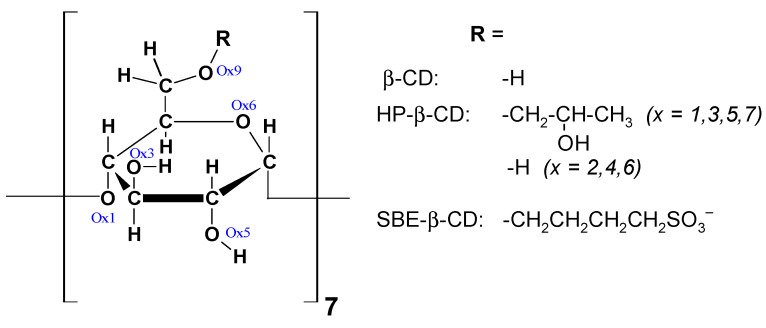
The numbering of selected atoms in CD molecules, taken from the β-CD crystal structure file (CCDC 2236679 [11]). x represents the number of a residue and ranges from 1 and 7. To ensure the three-character labelling of β-CD atoms, each of the ten hydrogen atoms belonging to the same glucose unit is designated by one digit, which means that the numbering is different from the commonly used notation in nuclear magnetic resonance studies.

CDs are a family of cyclic oligosaccharides, consisting of a macrocyclic ring of glucose subunits linked by α-1,4-glycosidic bonds (Scheme 2). The shape of CD molecules is similar to a truncated cone, with a hydrophilic surface and a hydrophobic inner cavity into which the hydrophobic drug molecules may enter. CDs differ in the number of glucopyranose units (six for α-CD, seven for β-CD, and eight for γ-CD) and, consequently, in the width of their narrow and wide rims. For most drug molecules, the capacity of β-CD (with seven glucose residues) is appropriate for the formation of the inclusion complex [8]; however, according to the European Medicines Agency (EMA), only a few CDs are approved as drug excipients [9]. β-CD can be used in oral but not parenteral formulations, while the hydroxypropyl (HP) and sulfobutyl ether (SBE) derivatives of β-CD (denoted as HP-β-CD and SBE-β-CD, respectively) have been approved for oral and parenteral use. Taking this into account, we selected three of the above-mentioned CDs for further calculations. Based on the review by Boczar and Michalska [10], by the end of 2021 there were 34 reports of cephalosporin antibiotics with various CDs, including β-CD, HP-β-CD, and SBE-β-CD. A significant increase in solubility was reported for cefuroxime axetil, cefixime, cefpodoxime proxetil, and cefdinir. Other advantages of CD complexation included modifying the drug-release profile, slowing down the degradation of the drug, improving biological membrane permeability, and enhancing antimicrobial activity [10].

Due to the random substitution of hydroxyl groups in HP-β-CD and SBE-β-CD which results in amorphism of these CDs, it is impossible to obtain single crystals of their inclusion complexes, and consequently to determine the structures using diffraction techniques. For them, computational techniques are the only way to gain insight into the most-likely three-dimensional geometries of complexes in solutions at different pH levels. Molecular dynamics (MD) is based on calculating the forces acting on each atom, taking into account the influence of neighbouring atoms. During this study, physical intermolecular interactions are analysed, which makes MD the appropriate technique for estimating the host–guest affinity, expressed as the Gibbs free energy (ΔG) of complex formation [8].

In general, cephalosporin antibiotics contain ionisable functional groups (carboxylic, amino, etc.), often more than one. The presence of the charges on them may affect the solubility of the drug and the formation of CD inclusion complexes. Consequently, the inclusion phenomena may occur differently at different pH values [12]. Meanwhile, the covalent bonds can neither form nor break during MD simulations, therefore the analysed drug molecule cannot adjust its protonation state to the selected solvent. This means that the covalent bonding structure has to be explicitly known before starting the simulation; however, most researchers perform their MD studies only with the neutral, non-ionised form of the drug, which consequently limits the applicability of the obtained results to pure aqueous, non-buffered solutions. A noteworthy exception was the recent work of Cirri et al. [13], who observed that the potential energies of the interaction between SBE-β-CD and cefixime differ by 75 kJ/mol (17.9 kcal/mol) depending on whether the charge of cefixime was 0 or −2. Nevertheless, in the case of ceftobiprole, currently there is no publicly available data on the protonation states and pK_a_ values of the constituent functional groups. Therefore, the MD simulations in this study were preceded by pK_a_ prediction with the use of specialised software, which made it possible to describe the CD complexation of ceftobiprole as an approximate function of pH, unlike similar works.

There are several scientific reports of CD complexes with cephalosporin antibiotics which contain, among others, theoretical studies. Gieroba et al. [14] investigated the complexes of cefuroxime axetil with α-, β-, γ-, and HP-β-CD (with six out of seven possible 2-OH groups substituted). Regardless of whether the initial structure was the complex or the separated host and guest within a distance of at least 1.5 nm, the inclusion complex was formed during the 500 ns of MD simulation with the use of the GROMOS force field. The obtained structures were similar to those obtained in the docking study. Next, the ΔGs of complex formation were calculated by the thermodynamic integration method, using 21 simulations with different coupling parameters λ, each lasting for 100 ns. Mizera et al. [15] found the thermodynamically-preferred structures of the complexes of cefuroxime axetil and cefetamet pivoxil together with seven CDs by using a three-step procedure consisting of (i) docking, (ii) 100 ps of MD with MMFF94, and (iii) geometry optimisation with the semi-empirical parametrised model 7. Standard enthalpies (ΔHs) of complex formation were determined based on the heats of formation of each ingredient. Other studies of cephalosporin/CD complexes generally involve the manual or docking-assisted building of the complex structure, followed by energy minimisation using a selected force field, and then calculating the binding energy or ΔH of formation [16,17,18,19]; however, MD studies are then omitted, and the obtained energy does not take into account solvation nor the change in the entropy of the system. Interestingly, it has also been proposed to calculate ΔG using the Generalised Born Surface Area (GBSA) equation applied directly to the structures predicted in the docking study, without performing MD simulations [20].

The aim of this study was to estimate the affinity of native ceftobiprole to the selected CDs (β-CD, HP-β-CD, and SBE-β-CD), which has not been investigated so far. The algorithm consisted of the following steps: (i) the models of substituted CDs were prepared, (ii) the pK_a_ values of functional groups in the ceftobiprole molecule were predicted, which allowed us to propose the dominant protonation states at different pH values, (iii) the initial structures of the complexes were obtained by docking, (iv) the systems were parametrised with the selected force field, the potential energy of the systems was minimised, and the systems were equilibrated to a constant temperature and pressure, (v) MD simulations were performed, and (vi) the results were analysed based on the obtained trajectories. The results were: the geometry of the complex (spatial arrangement of ceftobiprole and CD), the hydrogen bonding, and the ΔG of complex formation. Based on the obtained results, the most-preferential conditions of complex formation were determined. Then, the results obtained from theoretical studies were verified using high-performance liquid chromatography (HPLC) with UV detection.

## 2. Results and Discussion

### 2.1. Preparation of Models for Substituted CDs and pK_a_ Prediction for Ceftobiprole

In the first step, the single molecule models of HP-β-CD and SBE-β-CD were built in such a way as to largely mimic commercially-available CDs with an average degree of substitution (DS) of 4.2 and 6.5, respectively. The primary hydroxyl groups on each CD glucose unit (containing an Ox9 oxygen atom) were assumed to be the most reactive to derivatisation. Therefore, the SBE-β-CD structure with primary hydroxyl groups of all seven glucose units substituted with sulfobutyl groups (corresponding to DS = 7) was considered the most representative structure (Scheme 2). For the HP-β-CD, an alternating substitution pattern was used, in which HP substituents were added to Ox9 atoms in the first, third, fifth, and seventh glucose units, i.e., to O19, O39, O59, and O79 atoms, resulting in a DS = 4. Meanwhile, it is well-known that HP and SBE groups are very flexible due to the number of rotatable bonds, so it is very difficult (and impractical) to find a single conformer that minimises the potential energy surface. Therefore, a different approach was used, namely MD simulations of only the HP-β-CD and SBE-β-CD in water were performed to find the conformer with the largest radius of gyration. Such a conformer is characterised by the least steric hindrance for the drug molecule that can be incorporated into the CD cavity.

**Scheme 3:**
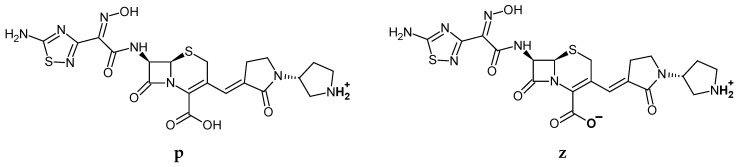
The predicted protonation states of ceftobiprole: protonated ion, hereinafter marked with the letter “p”, and zwitterion, hereinafter marked with the letter “z”.

Before starting the simulation, the structure of the covalent bonds of the studied molecules, including the protonation of all ionisable functional groups, should be thoroughly understood. At a certain pH value, ceftobiprole exists as a mixture of different forms, with some groups being protonated and others deprotonated. It is very important to correctly predict the protonation states of ceftobiprole, since the calculations relate to the thermodynamics in aqueous solutions where SBE-β-CD is negatively charged (total charge = −7); therefore, the electrostatic interactions between ceftobiprole and CD must be taken into account. Based on the calculated pK_a_ values of the ionisable functional groups, the dominant form at a given pH should be predicted; however, the use of different software leads to significantly different pK_a_ values. Based on the adopted pK_a_ results, multiple general conclusions can be drawn: the secondary amino group is protonated over a wide range of pH values (including acidic and neutral), and the pK_a_ of the carboxylic group is about 3 (3.00 by Chemicalize or 3.24 predicted by Epik 7), so at a low pH value (around 1–2), ceftobiprole should be present mainly as a cation, while at a higher pH, ceftobiprole can be expected to exist as a zwitterion (Scheme 3).

Another important conclusion from the pK_a_ calculation is that the primary amino group attached to the thiadiazole ring is not protonated but loses H^+^ at a high pH value (much greater than 7). This behaviour of the –NH_2_ substituent, which may seem surprising at first glance, can be explained by the electron-withdrawing properties of the aromatic thiadiazole ring, which destabilises the protonated form of an amine and consequently decreases the basicity of the –NH_2_ group. It is worth noting that similar results have been obtained in the fragmentation pattern analysis of the mass spectroscopic studies of the ceftobiprole molecule [21]. In the positive ion mode, protons were most easily attached via the secondary amino group, then the resulting ions lost their neutral fragments, ending up with only the pyrrolidine ring, which was the smallest fragment ion detected in the analysed fragmentation spectra. In contrast, the primary amino group did not easily attach a proton, and therefore, there were very few detectable ion fragments containing the aminothiadiazole ring.

### 2.2. Molecular Docking Study

Molecular docking is a well-known computational technique to predict the binding between a receptor (CD) and a ligand (drug) [22]. In our research, docking studies were performed for three different protonation states of ceftobiprole: the non-ionised molecule (denoted as 0), the protonated ion (p), and the zwitterion (z). Ligand-receptor affinities were calculated using the AD4 scoring function. As a result of each study, the program AutoDock Vina proposed the nine most-stable structures of the 1:1 complex, i.e., structures characterised with the greatest (negative) affinity.

**Scheme 4:**
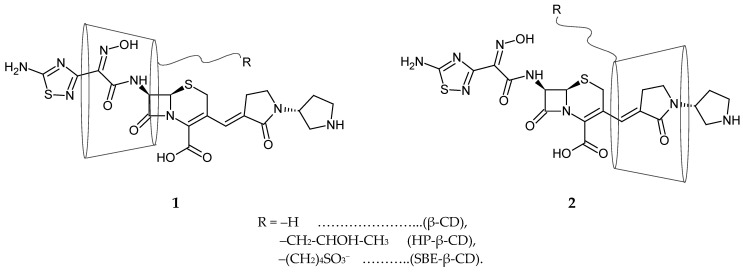
Two opposite directions of ceftobiprole molecule inside the CD cavity: (**1**) the wide rim of CD directed towards the aminothiadiazole ring; (**2**) the wide rim of CD directed towards the pyrrolidine ring.

For the HP-β-CD and SBE-β-CD, regardless of the protonation state, the most-negative affinity value was obtained for the structure with the wide rim of CD directed towards the aminothiadiazole ring (Table 1, Scheme 4)—this orientation is hereinafter referred to as “1”. Among the remaining structures, the one with the most-negative affinity for the ceftobiprole molecule directed oppositely from the inside of the CD cavity (Scheme 4), hereinafter referred to as “2”, was also taken as a starting point for an alternative MD analysis. This approach can be justified as follows [8]: in the timescale of the molecular dynamics simulation (here 100 ns), it is practically impossible for a drug molecule to escape the CD cavity and re-enter in the opposite direction.

### 2.3. MD Simulation and Analysis of Trajectories

In MD studies, there are several so-called general force fields, i.e., those parametrised for use with many different organic molecules, but not biomolecules (drug molecules belong to this group). In this study, a General AMBER Force Field (GAFF) was used together with an AMBER99SB because of their compatibility with the GBSA method. Complexes with the structure predicted in the docking studies were placed in a cubic box with water molecules.

**Figure 1:**
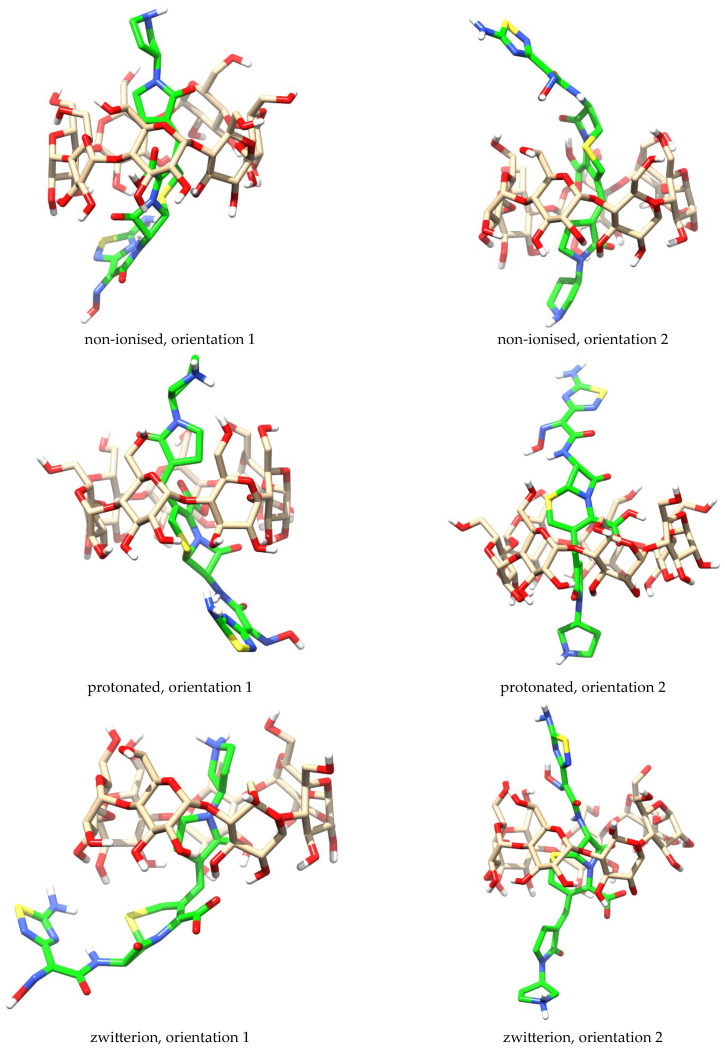
Central structures of ceftobiprole/β-CD complexes based on RMSD analysis.

**Figure 2:**
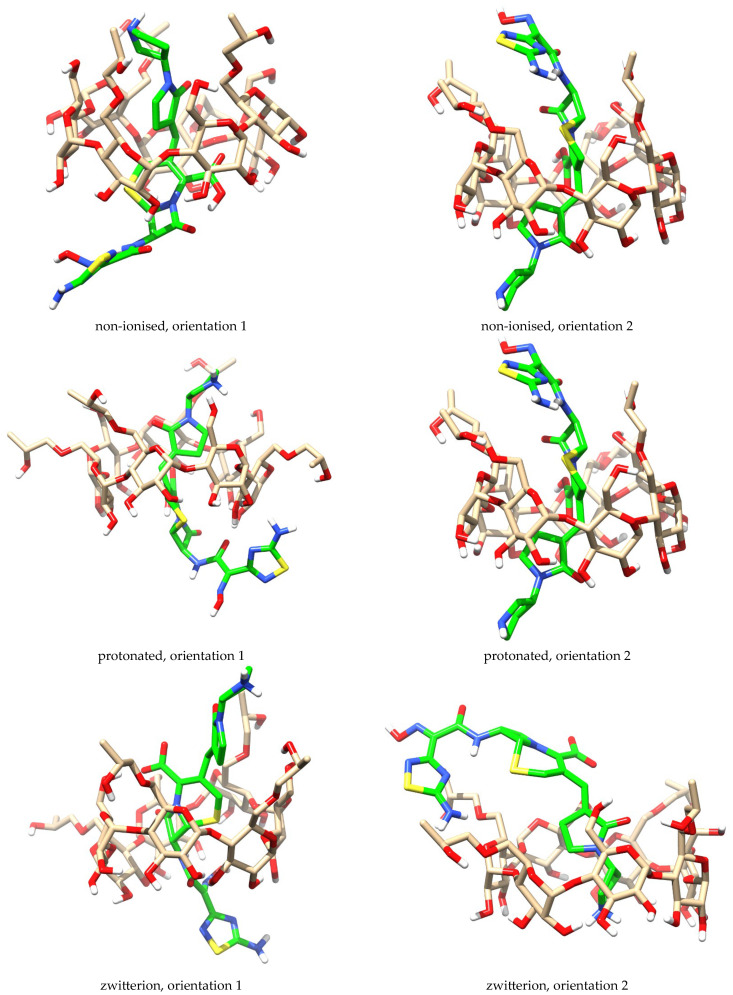
Central structures of ceftobiprole/HP-β-CD complexes based on RMSD analysis.

**Figure 3:**
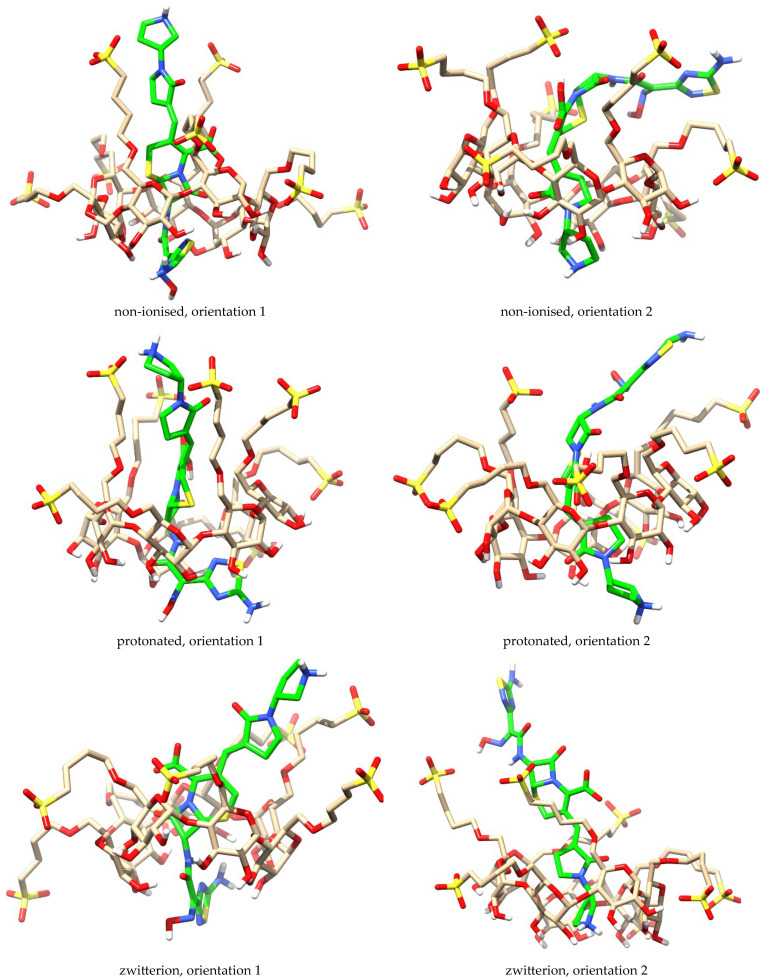
Central structures of ceftobiprole/SBE-β-CD complexes based on RMSD analysis.

After minimising the potential energy, all atoms were given initial velocities according to the Maxwell-Boltzmann distribution, and then, based on the calculated forces, the motions of all atoms were calculated. As a result of the frequent collisions with water molecules, the HP and SBE side chains in CDs constantly changed their conformations. For the complex of ceftobiprole z2 with the SBE-β-CD complex, the simulation time was extended to 160 ns because drift was observed in the energy plots. To identify the most-representative structure for each simulation, the root-mean-square deviation (RMSD) analysis of the atomic positions was performed. Briefly, the *gmx cluster* function with a cut-off value of 0.10 Å for β-CD, 0.11 Å for HP-β-CD, and 0.16 Å for SBE-β-CD was used to group snapshots representing similar complex structures into clusters. Each cluster possesses a particular number of snapshots, which corresponds to a certain fraction of the simulation time. The central structure is defined as the structure with the smallest average RMSD of all other structures of the cluster containing the majority of the snapshots (that is, of the cluster corresponding to the longest period of the simulation time). For all 18 simulations performed, the extent of inclusion of ceftobiprole into the CD cavity can be easily observed from the presented central structures, as shown in Figure 1 (β-CD), Figure 2 (HP-β-CD), and Figure 3 (SBE-β-CD). The PDB files containing these structures can be found in the Supplementary Materials.

A hydrogen bond (H-bond) analysis of the H-bonds between ceftobiprole and CD was performed using the *gmx hbond* command. This function identifies possible donors (in the case of zwitterion, this number decreases by one as the carboxylic group of ceftobiprole dissociates) and acceptors in the system, thus predicting the possible H-bonds that may emerge between ceftobiprole and CD. A geometrical criterion is verified for each timeframe. An H-bond is formed if the donor–acceptor distance is not greater than 0.35 nm, and the donor–hydrogen–acceptor angle is not less than 150°. The occupancy of a given H-bond is defined as the fraction of timeframes in which this criterion is fulfilled. For all performed simulations, Table 2 contains the average number of H-bonds detected per timeframe, and a list of donor–acceptor pairs for which the H-bond occupancy is not less than 10%.

### 2.4. GBSA Results

There are many methods for estimating the ΔG of host–guest complex formation [23], most of which are computationally very demanding. They require many simulations, e.g., for different degrees of guest inclusion in the CD cavity (umbrella sampling) or using many different values of the coupling parameter λ (thermodynamic integration). In this study, the GBSA method was used due to its very good computational efficiency, as this model requires only one trajectory of the complex, i.e., one simulation for each system. According to a recent review article [8], this method is widely-accepted in the field of CD inclusion complexes. The general ΔG consists of three parts: $\Delta E_{MM}$, which is the host–guest binding enthalpy independent of the solvent (including van der Waals and electrostatic terms), the enthalpy of solvation Δ*G_solv_* (described below), and the entropic term $-T\Delta S_{config}$, where $\Delta S_{config}$ is configurational entropy including the translational, rotational, and vibrational degrees of freedom. Based on the same trajectory, these terms are calculated first for the complex, and then for the host and the guest alone. The difference between them is given as a final result.

The total ΔG of solvation in the GBSA model is decomposed into an electrostatic (GB) and non-electrostatic (SA) part. The first is calculated as the free energy of first removing all charges in a vacuum, and then adding them again in the presence of a continuum solvent environment. It is assumed that the interior of the atom is filled with a material with a dielectric constant of ε = 1, while the molecule is surrounded by a solvent with ε = 78.5. Each atom in a molecule is represented as a sphere with a charge *q_i_* at its centre, and is characterised by the so-called effective Born radius, *R_i_*, which is calculated many times depending on the distance of the atom from the surface of the molecule. The non-electrostatic contribution is proportional to the total surface area available to the solvent and is due to the combined effect of the favourable van der Waals attraction between the solute and the solvent, and the unfavourable cost of breaking the solvent structure around the solute. The obtained ΔGs for the tested systems are presented in Table 1, while the contributions to the total ΔG values of individual terms are presented in Table 3 (β-CD), Table 4 (HP-β-CD), and Table 5 (SBE-β-CD). The values in kcal/mol can be converted to those in kJ/mol through multiplication by a factor of ca. 4.184.

As can be seen in Table 3, Table 4 and Table 5 the formation of complexes with ceftobiprole should always be a spontaneous process (ΔG < 0), taking into account the balance between the favourable enthalpic effect (physical interactions between ceftobiprole and CDs leading to a ΔH < 0) and the unfavourable entropic effect (ΔS < 0, i.e., –TΔS > 0, resulting from the decrease in the number of degrees of freedom in the system when ceftobiprole is immobilised in the CD cavity). Both the van der Waals and electrostatic interactions, as well as the SA terms, are negative, which means that they stabilise the formed complex, while the GB term is always positive. Exceptionally high values in both the electrostatic and GB terms are obtained with the cationic ceftobiprole with the anionic SBE-β-CD.

Some authors argue that there is no need to calculate the entropic term, as it should vary slightly between similar complexes. This simplification, which has not been applied here, would have the least impact on complexes with the HP-β-CD, for which the –TΔS ranges from 23.17 to 24.49 kcal/mol, while it does not apply at all in the case of SBE-β-CD, which possess seven flexible SBE chains (–TΔS ranging from 22.90 to 27.39 kcal/mol). Differences in the entropy result from the vibrational contribution, which can be positive or negative depending on the charges and orientation of the ceftobiprole.

Table 1 compares the affinities obtained from the molecular docking study (ranging from −5.91 to −7.75 kcal/mol) and the ΔG values obtained using the GBSA (from −3.50 to −12.62 kcal/mol). The ΔG of complex formation, calculated as the average over the simulation time, including multiple conformers, is a better measure of the interaction of CD and ceftobiprole in an aqueous solution than the result of docking to a rigid structure of one CD conformer. The discussed differences clearly explain why the structures obtained in the docking study were only used as starting structures for the MD/GBSA calculations.

Interestingly, the docking study predicted that the β-CD/ceftobiprole complexes with orientation 2 were thermodynamically favoured over orientation 1; however, the GBSA study found the opposite to be true. In all simulations with all three CDs, orientation 1 had a more negative ΔG, which means that the probability of finding a complex in orientation 1 is much higher than in orientation 2. According to the Boltzmann distribution, the ratio of the probabilities of two states, $p_{2}$/$p_{1}$, can be determined as follows:

(1) $$\frac{p_{2}}{p_{1}}=\exp\left(-\frac{\Delta E}{RT}\right)$$

where ΔE is the difference in energy of the analysed states in kcal/mol, R = 1.9872 cal mol^−1^ K^−1^ is the universal gas constant, and T = 298.15 K is the temperature of the system. Assuming that ΔE can be expressed as the difference between the ΔGs determined for orientations 1 and 2, which ranges from 0.55 to 4.95 kcal/mol for different systems, the probability of finding the complex in alignment 2 varies between 0.02 and 28%. The ΔG of complex formation is related to the stability constant *K* by means of the following equation:

(2) ΔG=−RTlnK

However, in the works on the MD of CD complexes, calculations of *K* based on the ΔG are hardly encountered. This approach, in which the exponent of the ΔG would be calculated, leads to an exponential exaggeration of any errors in the determination of the ΔG. Moreover, it would be difficult to compare the value calculated in this way with the experimental value, because the latter largely depends on the method and conditions of its determination [24]. On the other hand, it is safe to compare the ΔGs calculated using the same method. Based on Equation (2), the more negative the ΔG, the higher the *K*, which means that the equilibrium between the non-complexed molecules and the complex is more towards a complex formation. In other words, the probability of finding a complex instead of unbound molecules in a given solution would increase. The calculated values of the ΔG can be ordered in two ways: from the perspective of CD, or according to the protonation state of ceftobiprole (0, p, z). For β-CD, the stability of the complex decreases in the order of z > p > 0, for HP-β-CD, in the order of 0 > p > z, whereas for SBE-β-CD, it is in the order of p > 0 > z. From this different point of view, the preference for non-ionised ceftobiprole decreases as follows: HP-β-CD > SBE-β-CD > β-CD, and the order is slightly different for the cationic form, SBE-β-CD > HP-β-CD > β-CD, while in zwitterionic form the order is β-CD > HP-β-CD > SBE-β-CD. Consequently, for each CD there is a thermodynamically preferred protonation state of ceftobiprole with which the complex is most stable, and at certain pH values (at which ceftobiprole is characterised with a certain protonation state), there is a selected CD forming the most stable complexes. Of the 18 simulations performed, the most negative ΔG was obtained for the complex with cationic ceftobiprole and SBE-β-CD (−12.62 kcal/mol, which corresponds to −52.80 kJ/mol).

### 2.5. Comprehensive Analysis of the MD Results

The simultaneous analysis of the geometric structure of the complexes (Figure 1, Figure 2 and Figure 3), H-bonds (Table 2), and individual contributions to the ΔG (Table 3, Table 4 and Table 5) allows us to make several important observations. For neutral CDs (β-CD and HP-β-CD) together with non-ionised or positively charged ceftobiprole, the same fragment of the ceftobiprole molecule is inserted into the CD cavity, whether it is in alignment 1 or 2. This fragment contains four conjugated double bonds, from the C=O bond in the carboxylic group to the C=O bond in the 2-pyrrolidone ring, thus possibly allowing non-polar interactions with the hydrophobic interior of the CD cavity. H-bonds are formed between the hydroxyl groups of CDs and several oxygen atoms of ceftobiprole, including one in the 2-pyrrolidone ring (O2), the hydroxyl oxygen of the carboxylic group (O4), and in the amide bond linking the cephem group to the aminothiadiazole ring (O1). It can be hypothesised that the increased number of H-bonds formed by the HP-β-CD compared to those formed by the native β-CD is the probable cause of the higher stability of the HP-β-CD complexes with non-ionised and cationic ceftobiprole.

The nature of SBE-β-CD, involving its overall charge of −7, changes the mode of ceftobiprole inclusion compared to the smaller and neutral CDs. For orientation 1, with the aminothiadiazole ring protruding from the wide rim, the amide group is inserted into the CD cavity, whereas the cephem moiety and the five-member rings remain outside. Strong hydrogen bonds can be observed between the O1 atom of the ceftobiprole and the O15 atom of the SBE-β-CD. It is noteworthy that some SBE chains align parallel to the ceftobiprole molecule, allowing the SO_3_^−^ groups at the ends of these chains to interact with the ceftobiprole secondary amino group. This interaction is particularly strong in acidic solutions, in which this amino group carries a positive charge and is probably responsible for the greatest stability of this complex among all the performed simulations. On the other hand, for the complexes with arrangement 2, the 2-pyrrolidone ring is inserted into the CD cavity.

The structures of the complexes obtained with zwitterionic ceftobiprole differ significantly from those analysed so far, because the insertion of the negatively charged carboxylic anion into the hydrophobic CD cavity is not thermodynamically preferred. This suggests that the inclusion phenomena change when the complex is formed in a buffered solution of nearly neutral pH, compared to the previously discussed protonation states present in pure water and acidic solutions. Often it turned out that the equilibrium structure in the MD simulation was significantly different from that obtained in the docking study. In the case of β-CD, the 2-pyrrolidone ring is inserted into the CD cavity when the orientation of ceftobiprole is 1, and the β-lactam ring is inside the CD cavity when the orientation is 2. In both orientations, very strong H-bonds are formed with the carbonyl oxygen of the carboxyl group (O3) of ceftobiprole. The exceptionally high number of H-bonds per timeframe, 3.042 for orientation 1, is a possible reason why the β-CD complexes are most stable with the zwitterionic ceftobiprole compared to those in other protonation states. For both substituted CDs (HP- and SBE-β-CD), the inclusion depth of zwitterionic ceftobiprole is similar, but different from that observed with the native β-CD—in orientation 1, the β-lactam is inserted into the CD cavity, while in orientation 2, only the pyrrolidine ring is inserted whereas the rest of the molecule protrudes from the narrow rim. The GBSA calculations showed that the zwitterionic ceftobiprole forms the least-stable complexes with substituted CDs.

### 2.6. Experimental Verification of Theoretical Results Using HPLC

There are several experimental methods for studying CD inclusion complexes. Among them, HPLC was chosen, which allows for the analysis of changes in the physicochemical properties of the guest (such as the solubility and chemical stability) during the formation of the inclusion complex. For this purpose, the HPLC method developed and optimised by our research group was used [21].

First, the impact of CD addition on the peak area of ceftobiprole was investigated. The peak area of the ceftobiprole in water (0.04 mg/mL, corresponding to 0.08 mM) was compared with the peak area of the ceftobiprole of the same concentration obtained in an aqueous solution with the addition of the HP-β-CD or SBE-β-CD at a concentration of 10 mM. It was found that the area of the peak integrated at 320 nm (the first UV maximum of ceftobiprole absorption) increased only about 2% (despite a more than 100-fold molar excess of CD), while at 230 nm (the second UV maximum) no significant increase was observed in peak area, thus showing the equality of the response of ceftobiprole with and without CDs.

The impact of CDs on the solubility of ceftobiprole was then assessed. Such studies have not been performed for ceftobiprole so far, therefore, the time needed to obtain a saturated aqueous solution or the time needed to achieve complexation equilibrium were not known. Therefore, kinetic solubility studies (described in the Section 3) were performed. Supersaturated suspensions of ceftobiprole, with or without CDs, were sampled at specified time intervals and analysed using HPLC. The concentration of ceftobiprole obtained in this way, corresponding to a saturated solution, can be considered its maximum solubility under specified conditions. These values, changing over time, determined in three different solvents (water, 0.1 M HCOOH, and 0.1 M HCl), are summarized in Table 6. The relative increase in ceftobiprole concentration in saturated solution in the presence of CD was also provided. Due to the insufficient solubility of the β-CD in water and acidic solutions, tests were carried out only with substituted CDs.

In water, after 5 h of shaking, the solubility of ceftobiprole was 0.085 mg/mL (0.16 mM). In 10 mM of HP-β-CD, this value increased to 0.096 mg/mL (0.18 mM), i.e., by 13%, while in 10 mM of SBE-β-CD the solubility of ceftobiprole reached 0.116 mg/mL (0.22 mM), which means an improvement of 36%. In an acidic medium created to force the protonation of ceftobiprole, such as 0.1 M HCOOH (pH ≈ 2.3), after 24 h of shaking, the solubility of ceftobiprole was 0.146 mg/mL (0.27 mM). In 10 mM of HP-β-CD this value increased to 0.164 mg/mL (0.31 mM), i.e., by 13%, while in 10 mM of SBE-β-CD the solubility of ceftobiprole reached 0.224 mg/mL (0.42 mM), which means an improvement of 53%. In 0.1 M of HCl (pH ≈ 1), the concentration of ceftobiprole reached 0.987 mg/mL after 5 h of shaking. The obtained results showed that after a mixing (incubation) period of 24 h and more, the concentration of ceftobiprole constantly decreased, which indicated that the degradation processes outweighed the increase of the dissolution of ceftobiprole suspended in the solvent. After 24 h, the concentration of ceftobiprole in 0.1 M of HCl was 0.938 mg/mL, while in 10 mM of HP-β-CD it was 1.012 mg/mL (+8%), and in 10 mM of SBE-β-CD it reached 1.384 mg/mL (+48%). In summary, it can be concluded that the SBE-β-CD improves the solubility of ceftobiprole to a greater extent than the HP-β-CD, both in neutral and acidic solutions.

How do these results support those obtained from theoretical studies? It is intriguing why the solubility of ceftobiprole, even in more than a 50-fold excess CD, increases relatively little. To answer this question, it may be useful to analyze the predicted structures of the complexes shown in Figure 1, Figure 2 and Figure 3. The ceftobiprole molecule consists of five connected rings, two of which form the bicyclic cephem moiety. It is clear from the analysis of Figure 1 that the macrocyclic ring of the CD molecule surrounds a short segment of the ceftobiprole molecule. As a result, a major part of the ceftobiprole molecule is still exposed to direct contact with water molecules, which is probably thermodynamically unfavorable. It can be anticipated that the relatively small partial improvement in ceftobiprole solubility is probably due to the protection of only part of the ceftobiprole molecule by the macrocyclic CD ring.

It therefore remains to explain why the improvement in the solubility of ceftobiprole in unacidified water was greater for the SBE-β-CD than for the HP-β-CD, even though the calculated stability constant was found to be higher for the latter. It can be assumed that the decisive factor is not the *K* value, but the geometrical shape of the complex. Taking into account the high *K* value, obtained in theoretical calculations for all the tested complexes, as well as an approximately 50-fold molar excess of CD in experimental studies, it can be stated that almost 100% of ceftobiprole molecules are complexed by CDs. Meanwhile, a comparison of the corresponding structures in Figure 2 and Figure 3 shows significant differences in the shapes of the complexes. In the case of the SBE-β-CD, another part of the ceftobiprole molecule entered the CD cavity, and the SBE chains were arranged parallel to the ceftobiprole molecule, thus protecting it from direct contact with water.

The relatively low chemical stability of ceftobiprole was discussed in [21] and the kinetics of its degradation was determined. Based on the literature data on the improvement of the chemical stability of β-lactam antibiotics after their complexation with CDs [10,25], it was assumed that the inclusion of the ceftobiprole molecule in the CD cavity would protect the ceftobiprole molecule against hydrolysis in an acidic environment. Therefore, the kinetics of ceftobiprole degradation in two solvents was examined: in water at 45 °C and in 0.1 M of HCl at 25 °C, and the corresponding rate constants *k* were determined in the presence and absence of CDs. The reason for increasing the temperature was to obtain an appropriate degradation rate that would allow for an unambiguous determination of *k*. The obtained results are summarized in Table 7.

**Scheme 5:**
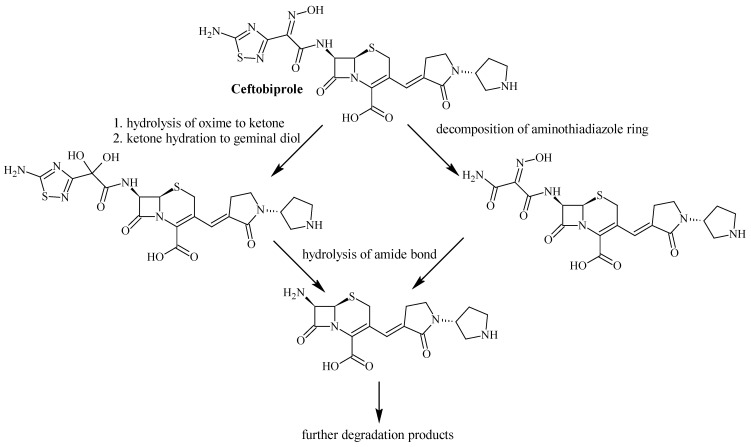
Degradation pathway under acidic conditions, proposed in work [21].

The results showed that the HP-β-CD accelerated the degradation of ceftobiprole under neutral conditions and inhibited the degradation under acidic conditions. This is consistent with the statement of Popielec and Loftsson regarding the influence of CDs on the chemical stability of drugs [25]. The authors indicated that the catalytic effect of CDs can be explained by the fact that CDs are oligosaccharides and, like other saccharides (e.g., glucose), catalyze the degradation of β-lactam antibiotics in aqueous solutions under neutral and alkaline conditions. The significant effect of the SBE-β-CD complexation on the stability of ceftobiprole in acidic conditions can be explained based on the knowledge of the structure of acid degradation products determined in the paper [21]. The degradation product that is formed first is the result of the hydrolysis of the oxime group to the keto group (Scheme 5). In the complex with the SBE-β-CD, the solvent access to the oxime group is limited because it is hidden in the CD cavity.

## 3. Materials and Methods

### 3.1. Computations

The structure of the ceftobiprole molecule was obtained from PubChem (CID 135413542 [26]). The pK_a_ values of functional groups were predicted using various software, including Chemicalize from ChemAxon (Budapest, Hungary) [27] and Epik 7, a part of the Schrödinger 2023-2 package [28]. The structure of the β-CD was taken from the Cambridge Crystallographic Data Centre (CCDC, deposition number 2236679 [11]). The structures of the substituted CDs (Scheme 2) were created manually, based on the crystallographic structure of native β-CD. For the HP-β-CD, the HP groups were attached to four selected primary hydroxyl groups (in the first, third, fifth, and seventh glucose unit), whereas for the SBE-β-CD, primary hydroxyl groups in all seven glucose units were substituted with SBE moiety.

The molecular docking studies were performed with the use of AutoDock Vina 1.2.3 [29,30], using AutoDock 4 force field, scoring function AD4, and computational exhaustiveness 32. As a result, nine structures of the complexes characterised by most negative affinity (expressed in kcal/mol) were proposed by the software. Two conformations were chosen out of them for the MD studies—one with the most negative affinity, and another one corresponding to the thermodynamically most-favoured complex with ceftobiprole directed oppositely inside the CD cavity. The resultant files were converted to the Protein Data Bank (.pdb) file format, and then the missing hydrogen atoms of the ceftobiprole molecule were added with use of Avogadro 1.2.0 [31].

In order to parametrise the complexes with the use of General AMBER Force Field (GAFF), the following parts of the AmberTools 20 suite [32] were applied separately for the ceftobiprole and CDs: antechamber [33,34] to calculate partial charges for each atom on the AM1/BCC level of theory, parmchk2 to obtain the missing parameters of GAFF, and LEaP to generate AMBER topologies. Then, AMBER topology files were converted to GROMACS files with the use of acpype 2022.6.6 script [35], separately for ceftobiprole and for CD.

MD simulations were performed in GROMACS 2022.5 [36]. Topology files were created manually based on the results obtained with acpype. Afterwards, cubic periodic boxes were created around the systems and filled with TIP3P water molecules. For the first tested system with the SBE-β-CD, which is the largest CD in our calculation, the dimensions of the box were selected so that the distance of the solute from the wall was at least 1.2 nm (greater than the longest cut-off), which corresponded to the edge of the box at 5.293 nm. In the subsequent studies with SBE-β-CD, the initial box sizes were set at the same value of 5.293 nm, while for the complexes with β-CD and HP-β-CD, the box sizes were set to 5.270 nm, leading to an almost equal number of solvent molecules (4701–4723) in all 18 systems. An appropriate number of sodium cations or chloride anions was added to the solvent as counter ions to neutralise the charge of the system. The corresponding numbers of the molecules in the systems are summarised in Table 8.

The potential energy of the systems was minimised using the steepest decent method. Afterwards, 100 ps of equilibration in NVT ensemble and 100 ps in NPT ensemble were performed. Position restrains were put on non-hydrogen atoms of the solute only during the equilibration. Then, 100 ns of production run was performed with the set of parameters chosen based on the GROMACS tutorial [37] and parameters proposed by acpype, listed below. The detailed description of the applied parameters of simulation can be found, for example, in the GROMACS tutorial [37] and manual [38]. The equations of motion were solved using leap-frog integrator with a time step of 2 fs, and with the use of the LINCS holonomic constraint algorithm applied for bonds to hydrogen atoms. A Verlet list of short-range neighbours was used. For van der Waals forces, a switching function at distances between 0.9 and 1.1 nm was applied (according to the output of acpype). The Particle Mesh Ewald (PME) method was used to calculate long-range electrostatic forces. Solute and solvent were coupled to two separate V-rescale thermostats at 298.15 K. The pressure of the system (1 bar) was controlled by Parrinello-Rahman barostat. Snapshots of the system were saved in 10 ps intervals.

The ΔG of complex formation was estimated using a GBSA equation [39] with the use of gmx_MMPBSA 1.6.1 software [40], based on AMBER’s MMPBSA.py 16.0 [41] and AmberTools 20 [31]. The calculations were performed with default parameters of gmx_MMPBSA 1.6.1, except for the concentration of ions (*saltcon* and *nmode_istrng*), which was set to 0.1 M for the systems with charged ceftobiprole. The modified model developed by Onufriev et al. (*igb* = 5) with the mbondi2 set of radii (PBRadii = 3) [42] was used. The entropic contribution was estimated by means of normal mode analysis for every tenth snapshot.

The Supplementary Materials contains the files with the applied commands, GROMACS topology, as well as the files with parameters for the energy minimisation, NVT and NPT equilibration, production run, and GBSA calculations.

### 3.2. HPLC Studies

The ceftobiprole was purchased from BenchChem (Austin, TX, USA). HP-β-CD (DS = 4.2, product number CY-2005.2) and the SBE-β-CD (DS = 6.5, product number CY-2041.2) was bought from CycloLab (Budapest, Hungary).

The deionised water was obtained from a Direct-Q 3 UV Millipore by Merck (Darmstadt, Germany). The acetonitrile (ACN, gradient grade for HPLC, ≥99.9%) was obtained from Honeywell (Seelze, Germany), the hydrochloric acid (HCl, pure p.a., 35–38%) was bought from POCH (Gliwice, Poland), the ammonium acetate (reag. Ph. Eur., ≥98.0%) was purchased from Merck (Darmstadt, Germany), the acetic acid (pharma grade, 99.9%) was obtained from AppliChem (Darmstadt, Germany), and the formic acid (HCOOH, reag. Ph. Eur., ≥98%) was obtained from Sigma-Aldrich (Darmstadt, Germany).

The supersaturated suspensions of ceftobiprole were prepared and sonicated for 10 min: 0.25 mg/mL in water, 0.50 mg/mL in 0.1 M of HCOOH and 2.00 mg/mL in 0.1 M of HCl. The appropriately weighed amounts of the selected CDs were then transferred to stoppered flasks: the HP-β-CD to the first one and the SBE-β-CD to the second one, while the third flask did not contain CDs and served as a reference. A volume of 6.5 mL of ceftobiprole suspension was added to each flask, while the concentrations of the selected CDs were 10 mM, respectively. The flasks were shaken at 750 rpm and 1 mL samples were taken from the flasks at intervals of 15, 150, and 300 min and then 24 and 48 h. The samples were centrifuged at 14,000 rpm for 10 min, and then the clear supernatants were analyzed using HPLC. For comparison, the standard solutions of ceftobiprole were prepared with a concentration below the solubility limit in each solvent. To determine the concentration of ceftobiprole in the sample, the peak area in the test sample was compared to the peak area of ceftobiprole in a standard solution of a known concentration.

In the kinetic experiments, the peak area of ceftobiprole was monitored as a function of time at 1.5 h intervals for 24 h (water) or 15 h (0.1 M of HCl). Pseudo-first-order kinetics was assumed and the rate constant k was determined as the slope of the line fitted according to the following equation:

(3) $$\ln\left(\frac{a}{a_{0}}\right)=-kt$$

where $a_{0}$ is the initial peak area of the ceftobiprole, and a is the peak area of the ceftobiprole at time t.

The HPLC analysis was performed using a Shimadzu Nexera-i LC-2040C (Kioto, Japan) liquid chromatograph with a UV–VIS detector, equipped with LabSolutions 5.87 data processing software. A chromatographic method developed in [21] was applied.

## 4. Conclusions

The interest in CDs in combination with drugs is constantly growing, and this trend will continue into the coming years due to their versatility and fascinating ability to improve pharmaceutical formulations in the design of innovative therapies. Among the many methods of studying CD complexes, computational techniques have gained great attention, as they may be used to predict the most likely geometries of complexes instead of utilising diffraction techniques. Although some molecular modelling analyses of CD complexes with cephalosporin antibiotics have been performed to date, few of them include a detailed study of the factors influencing the complex formation.

In this study, MD simulations were performed for 18 combinations of CD, and ceftobiprole protonation states and orientations. Unlike similar papers, this MD study additionally involves an attempt to predict the pK_a_ values for ionisable functional groups, which allows for proposing the dominant protonation states of ceftobiprole at different pH values. Consequently, the obtained results show how the inclusion phenomena change with pH. The analysis of the obtained MD trajectories included the spatial arrangement of ceftobiprole and CD, H-bonds forming between them, as well as energetic contributions to the total ΔG of complex formation. The complex formation with ceftobiprole proved to be a spontaneous process. Both the van der Waals and electrostatic interaction terms in the GBSA calculations were negative, which means that they stabilized the resulting complex. The differences in entropy were due to the vibrational contribution, which could be positive or negative depending on the charges and orientation of the ceftobiprole. The ΔG of complex formation involving multiple conformers was found to be a better measure of the interaction between ceftobiprole and CD in an aqueous solution than the result of docking to a rigid structure of a single CD conformer. In all simulations with all three CDs, orientation 1 had a more negative ΔG, meaning that the probability of finding the complex in orientation 1 was much higher than in orientation 2. Finally, three pairs consisting of the CD and the appropriate protonation states of ceftobiprole of the greatest stability have been found: β-CD and zwitterionic ceftobiprole, HP-β-CD and neutral ceftobiprole, and SBE-β-CD and protonated ceftobiprole. Out of them, the complex consisting of the SBE-β-CD and protonated ceftobiprole proved to be the most stable (ΔG = −12.62 kcal/mol = −52.80 kJ/mol) due to the electrostatic interactions between the anionic host and the cationic guest.

The obtained theoretical results were experimentally verified using HPLC. For the most preferential conditions of complex formation, in an acidic solution of ceftobiprole and SBE-β-CD, a significant effect of CD on the physicochemical properties of ceftobiprole was observed. The solubility (concentration of ceftobiprole in a saturated solution) increased ca. 1.5-fold, and the degradation rate constant of ceftobiprole decreased ca. 2.5-fold in 0.1 M of HCl. Similar effects have been reported in the literature for other cephalosporin antibiotics, so it can be concluded that these experimental results confirm theoretical predictions.

## Figures and Tables

**Table 1:** Summary of obtained values for affinity (docking study) and ΔG (GBSA) in kcal/mol.

Protonation States of Ceftobiprole	Arrangement of the Ceftobiprole Molecule in the CD Cavity	β-CD		HP-β-CD		SBE-β-CD	
		Docking	GBSA	Docking	GBSA	Docking	GBSA
0	1	−5.95	−6.97	−6.75	−12.27	−7.34	−8.89
	2	−6.96	−3.50	−6.38	−10.89	−6.84	−7.00
p	1	−5.91	−7.08	−6.38	−11.76	−7.73	−12.62
	2	−6.75	−5.05	−5.94	−6.49	−7.27	−7.71
z	1	−5.94	−11.44	−6.26	−6.73	−7.75	−5.88
	2	−6.83	−6.49	−6.16	−6.18	−6.66	−3.84

**Table 2:** H-bond analysis. Atom numbering is shown in Scheme 1 for ceftobiprole and Scheme 2 for CDs. *n*—the average number of H-bonds per timeframe. 0, p, z—different protonation states of ceftobiprole (Scheme 3). 1, 2—the arrangement of the ceftobiprole molecule in the CD cavity, presented in Scheme 4.

		β-CD		HP-β-CD		SBE-β-CD	
		26 (0, p)/25 (z) Donors, 49 Acceptors		26 (0, p)/25 (z) Donors, 53 Acceptors		19 (0, p)/18 (z) Donors, 70 Acceptors	
		*n*	Most Frequent H-Bonds in the Form: CD Atom–Ceftobiprole Atom (Occupancy)	*n*	Most Frequent H-Bonds in the Form: CD Atom–Ceftobiprole Atom (Occupancy)	*n*	Most Frequent H-Bonds in the Form: CD Atom–Ceftobiprole Atom (Occupancy)
0	1	1.999	all below 10%	2.288	O35-O1 (68.2%), O33-O1 (48.5%), O39-N1 (30.7%), O69-O2 (25.9%), O45-O3 (20.4%), O45-O4 (12.0%)	1.225	O15-O1 (58.3%)
	2	1.123	O75-O2 (10.6%)	1.789	O61-O4 (50.4%), O25-O2 (26.6%), O76-N2 (26.3%)	1.405	O65-N1 (17.5%), O75-O2 (16.5%), O(SO_3_^−^)-O4 (11.4%)
p	1	1.983	O41-O4 (12.4%), O61-O4 (11.9%), O51-O4 (10.7%)	2.522	O35-O1 (54.9%), O33-O1 (50.1%), O59-O2 (29.8%), O25-O1 (21.6%), O23-O1 (18.7%)	1.545	O25-O1 (56.2%), O53-O1 (10.6%)
	2	1.613	O75-O2 (16.0%), O41-O4 (16.7%), O65-O2 (12.5%), O55-O2 (11.4%), O31-O4 (10.8%), O21-O4 (10.5%)	1.436	O35-O2 (45.2%), O63-O2 (14.7%)	1.427	O65-O2 (40.3%), O23-O2 (22.7%)
z	1	3.042	O73-O3 (63.2%), O75-O3 (62.3%), O29-N1 (20.5%), O33-O2 (17.3%), O59-N1 (13.6%), O63-O4 (10.6%)	2.299	O69-O4 (71.5%), O13-O1 (28.1%), O43-O5 (19.9%), O55-O1 (10.5%)	1.605	O65-O1 (37.7%), O55-O1 (26.3%), O35-N2 (11.6%), O25-N2 (10.7%)
	2	2.106	O65-O3 (22.3%), O55-O3 (15.0%), O75-O3 (14.8%), O73-O3 (13.0%), O63-O3 (11.1%)	1.464	O69-O3 (37.9%), O76-N2 (17.6%), O15-N1 (15.0%), O29-O3 (11.9%)	0.909	O55-N1 (15.5%), O45-N1 (11.8%)

**Table 3:** Comparison of enthalpic and entropic terms contributing to the total ΔG of the formation of a complex with β-CD. 0, p, z—different protonation states of ceftobiprole (Scheme 3). 1, 2—arrangement of the ceftobiprole molecule in the CD cavity, presented in Scheme 4.

		β-CD					
		0		p		z	
		1	2	1	2	1	2
ΔH	van der Waals	−39.07	−37.39	−38.47	−36.56	−29.21	−35.45
	electrostatic	−18.19	−13.36	−26.26	−23.83	−66.72	−34.57
	Δ*E_MM_*	−57.26	−50.75	−64.73	−60.38	−96.62	−70.02
	GB	31.98	29.46	38.38	35.83	65.38	46.11
	SA	−4.21	−4.03	−4.24	−3.94	−3.90	−3.83
	Δ*G_solv_*	27.77	25.43	34.14	31.98	61.48	42.29
	total	−29.48	−25.32	−30.60	−28.49	−35.44	−27.74
−TΔS	translational	12.98	12.98	12.98	12.98	12.98	12.98
	rotational	11.12	11.14	11.12	11.14	11.07	11.18
	vibrational	−1.59	−2.30	−0.59	−0.68	−0.04	−2.92
	total	22.51	21.82	23.51	23.44	24.00	21.25
ΔG	total	−6.97	−3.50	−7.08	−5.05	−11.44	−6.49

**Table 4:** Comparison of enthalpic and entropic terms contributing to the total ΔG of the formation of a complex with HP-β-CD. 0, p, z—different protonation states of ceftobiprole (Scheme 3). 1, 2—the arrangement of the ceftobiprole molecule in the CD cavity, presented in Scheme 4.

		HP-β-CD					
		0		p		z	
		1	2	1	2	1	2
ΔH	van der Waals	−41.37	−44.93	−38.22	−40.94	−41.60	−38.35
	electrostatic	−27.02	−19.41	−35.96	−25.91	−31.77	−32.13
	Δ*E_MM_*	−68.39	−64.34	−74.18	−66.84	−73.37	−70.48
	GB	37.39	33.65	42.81	40.53	46.61	45.13
	SA	−4.44	−4.67	−4.40	−4.41	−4.41	−4.27
	Δ*G_solv_*	32.95	28.97	38.41	36.13	42.20	40.86
	total	−35.44	−35.37	−35.77	−30.72	−31.17	−29.62
−TΔS	translational	13.03	13.03	13.03	13.03	13.03	13.03
	rotational	11.19	11.22	11.22	11.22	11.30	11.17
	vibrational	−1.05	0.24	−0.24	−0.03	0.12	−0.76
	total	23.17	24.49	24.01	24.23	24.45	23.44
ΔG	total	−12.27	−10.89	−11.76	−6.49	−6.73	−6.18

**Table 5:** Comparison of enthalpic and entropic terms contributing to the total ΔG of the formation of a complex with SBE-β-CD. 0, p, z—different protonation states of ceftobiprole (Scheme 3). 1, 2—the arrangement of the ceftobiprole molecule in the CD cavity, presented in Scheme 4.

		SBE-β-CD					
		0		p		z	
		1	2	1	2	1	2
ΔH	van der Waals	−50.81	−44.74	−54.24	−46.09	−47.62	−40.81
	electrostatic	−5.00	−22.39	−261.01	−211.84	−14.81	−15.26
	Δ*E_MM_*	−55.82	−67.13	−315.25	−257.93	−62.44	−56.07
	GB	28.27	40.47	281.02	229.52	36.90	33.72
	SA	−5.19	−4.98	−5.78	−4.99	−4.68	−4.38
	Δ*G_solv_*	23.08	35.50	275.25	224.54	32.22	29.34
	total	−32.74	−31.64	−40.01	−33.40	−30.22	−26.74
−TΔS	translational	13.12	13.12	13.12	13.12	13.12	13.12
	rotational	11.35	11.35	11.35	11.38	11.36	11.32
	vibrational	−0.63	0.17	2.92	1.18	−0.15	−1.54
	total	23.85	24.64	27.39	25.68	24.33	22.90
ΔG	total	−8.89	−7.00	−12.62	−7.71	−5.88	−3.84

**Table 6:** Ceftobiprole concentration in water, 0.1 M HCOOH, and 0.1 M HCl at specified time intervals.

	Without CD	With HP-ß-CD		With SBE-ß-CD	
Time	c (mg/mL)	c (mg/mL)	Relative Increase	c (mg/mL)	Relative Increase
Ceftobiprole in water					
15 min	0.087	0.090	1.04	0.112	1.29
150 min	0.087	0.095	1.09	0.115	1.33
300 min	0.085	0.096	1.13	0.116	1.36
24 h	0.093	0.101	1.08	0.119	1.27
48 h	0.107	0.109	1.03	0.125	1.18
Ceftobiprole in 0.1 M HCOOH					
15 min	0.139	0.151	1.09	0.216	1.55
150 min	0.142	0.157	1.10	0.226	1.59
300 min	0.153	0.167	1.09	0.224	1.46
24 h	0.146	0.165	1.13	0.224	1.53
48 h	0.157	0.181	1.15	0.233	1.49
Ceftobiprole in 0.1 M HCl					
15 min	0.972	0.974	1.00	1.337	1.37
150 min	0.955	0.973	1.02	1.347	1.41
300 min	0.987	1.005	1.02	1.375	1.39
24 h	0.938	1.012	1.08	1.384	1.48
48 h	0.895	1.002	1.12	1.311	1.47

Relative increase—the ratio of ceftobiprole concentrations with and without CD.

**Table 7:** Comparison of rate constants *k* for ceftobiprole degradation.

	Water, 45 °C	0.1 M HCl, 25 °C
without CD	3.72 × 10^−6^ s^−1^	2.05 × 10^−6^ s^−1^
with 10 mM HP-β-CD	4.17 × 10^−6^ s^−1^	1.33 × 10^−6^ s^−1^
with 10 mM SBE-β-CD	1.31 × 10^−6^ s^−1^	0.81 × 10^−6^ s^−1^

**Table 8:** The numbers of water molecules and ions in the performed simulations.

		β-CD		HP-β-CD		SBE-β-CD	
		H_2_O	Ions	H_2_O	Ions	H_2_O	Ions
0	1	4720	--	4707	--	4707	7 Na^+^
	2	4723	--	4705	--	4712	7 Na^+^
p	1	4719	1 Cl^−^	4705	1 Cl^−^	4709	6 Na^+^
	2	4721	1 Cl^−^	4704	1 Cl^−^	4711	6 Na^+^
z	1	4723	--	4707	--	4709	7 Na^+^
	2	4722	--	4701	--	4708	7 Na^+^

## Data Availability

Data are contained within the Supplementary Materials.

## Supplementary Materials

The supporting information can be downloaded at: https://www.mdpi.com/article/10.3390/ijms242316644/s1.
